# Exercising your fat (metabolism) into shape: a muscle-centred view

**DOI:** 10.1007/s00125-020-05170-z

**Published:** 2020-06-12

**Authors:** Anne Gemmink, Patrick Schrauwen, Matthijs K. C. Hesselink

**Affiliations:** Department of Nutrition and Movement Sciences, NUTRIM School for Nutrition and Translational Research in Metabolism, Maastricht University Medical Centre+, 6200 MD Maastricht, the Netherlands

**Keywords:** Athletes, Exercise, Fat metabolism, Intramyocellular lipid droplets, Lipid droplet–mitochondria interaction, Lipid-droplet turnover, Liver, Muscle, Review, Type 2 diabetes

## Abstract

Fatty acids are an important energy source during exercise. Training status and substrate availability are determinants of the relative and absolute contribution of fatty acids and glucose to total energy expenditure. Endurance-trained athletes have a high oxidative capacity, while, in insulin-resistant individuals, fat oxidation is compromised. Fatty acids that are oxidised during exercise originate from the circulation (white adipose tissue lipolysis), as well as from lipolysis of intramyocellular lipid droplets. Moreover, hepatic fat may contribute to fat oxidation during exercise. Nowadays, it is clear that myocellular lipid droplets are dynamic organelles and that number, size, subcellular distribution, lipid droplet coat proteins and mitochondrial tethering of lipid droplets are determinants of fat oxidation during exercise. This review summarises recent insights into exercise-mediated changes in lipid metabolism and insulin sensitivity in relation to lipid droplet characteristics in human liver and muscle.

**Electronic supplementary material:**

The online version of this article (10.1007/s00125-020-05170-z) contains a slideset of the figures for download, which is available to authorised users.

**Figure:**
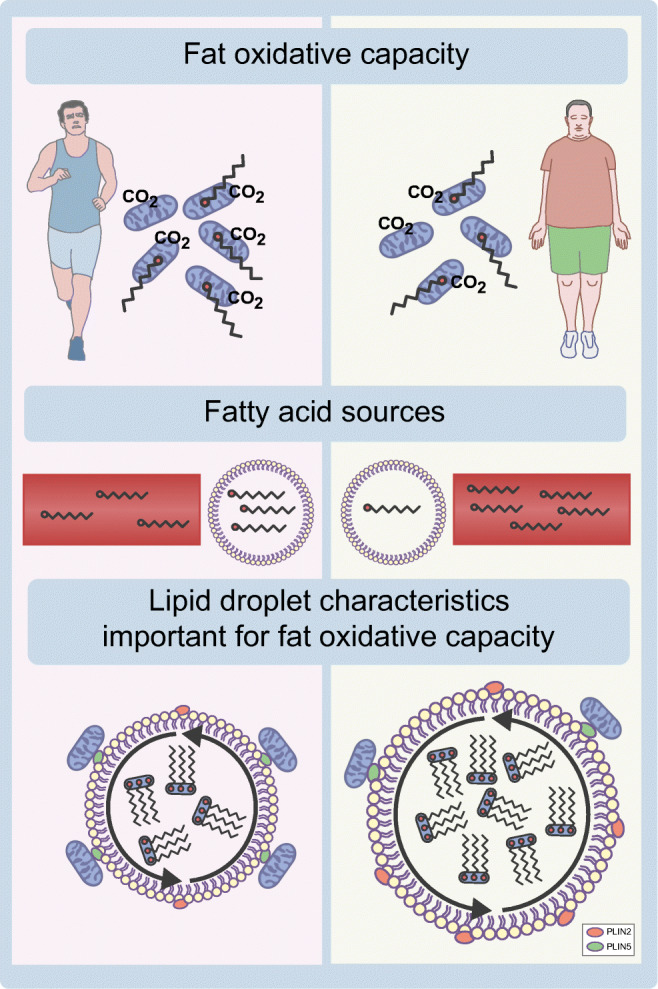
Graphical abstract

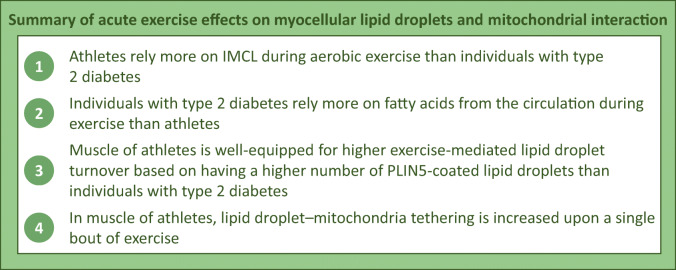

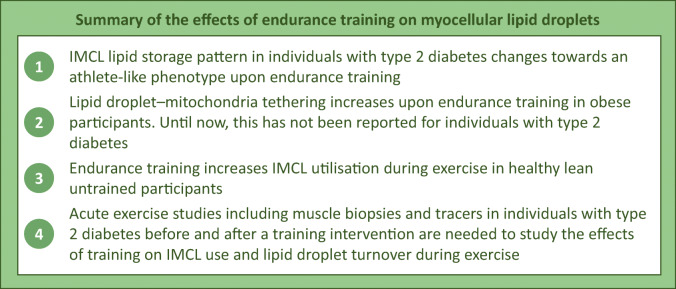

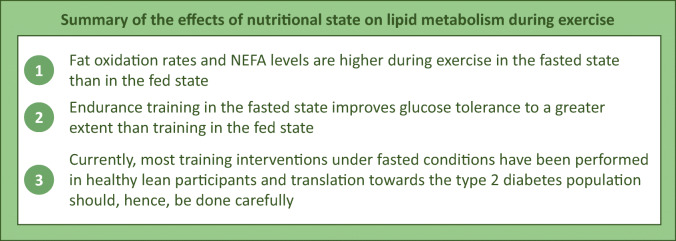

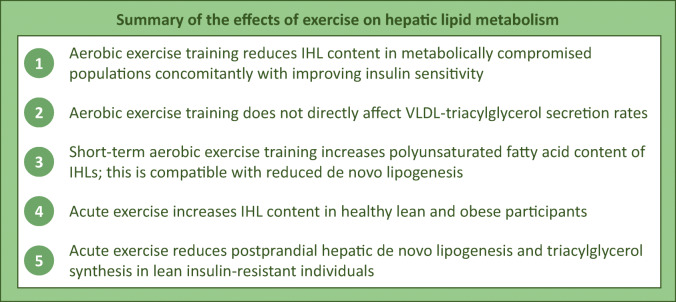









## Introduction

During physical exercise, the increase in energy demand is fuelled by oxidation of glucose and fatty acids [1]. The relative and absolute contribution of glucose or fat oxidation is dependent on the prandial state (and substrate availability), exercise intensity and training status [2]. Endurance-trained athletes are at the high end of the spectrum of fat oxidative capacity, whereas insulin-resistant individuals typically possess compromised fat oxidative capacity. In both populations, endurance training improved fat oxidative capacity. Fatty acids used during exercise can originate from the circulation, packed in triacylglycerol-rich particles originating from the liver or as NEFAs, predominantly originating from adipose tissue lipolysis [1]. The relative contribution of the different fat pools to whole-body fat oxidation is exercise-intensity dependent, with fat oxidation rising between 40% and 55% of maximal power output (*W*_max_) and then declining towards 75% *W*_max_. Tracer studies revealed that this comprises a drop in oxidation of NEFAs and triacylglycerol fat sources (intramyocellular lipid [IMCL] and lipoprotein-derived triacylglycerols) [3].

Other sources of exercise-fuelling fatty acids are the IMCLs, of which the majority is stored in triacylglycerol-rich lipid droplets dispersed throughout the muscle [1]. The observation that in non-athletes, insulin sensitivity correlates negatively with IMCL content led to the suggestion that IMCL content directly impedes insulin sensitivity. In contrast, trained athletes store IMCL to a similar level as insulin-resistant individuals, while being highly insulin sensitive; a phenomenon referred to as the ‘athlete’s paradox’ [4]. More recently, however, size, number, subcellular distribution and mitochondrial tethering of lipid droplets, as well as their decoration with lipid droplet coat proteins, appear to be discriminating determinants of fat oxidative capacity in an insulin sensitivity-dependent fashion [5, 6].

The aim of this review is to summarise recent insights into exercise-mediated changes in lipid metabolism and insulin sensitivity in relation to lipid droplet characteristics in human liver and muscle.

## Effects of acute exercise on lipid metabolism in human skeletal muscle

### IMCL utilisation during endurance exercise

**Fig. 1:**
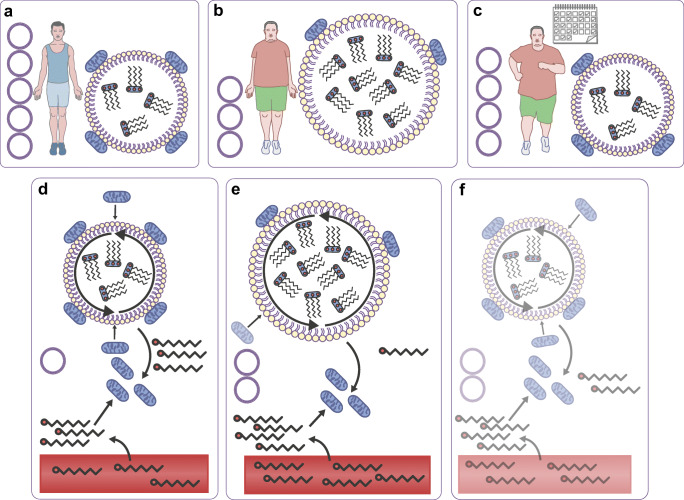
Skeletal muscle lipid metabolism: acute exercise and endurance training effects. Healthy active/endurance-trained athletes have IMCL content stored in many small lipid droplets (**a**). Contrarily, in people who are metabolically compromised (i.e. obese and type 2 diabetic individuals), the same amount of IMCL is stored in fewer, but larger lipid droplets (**b**). Triacylglycerols are shown within the lipid droplets. Lipid droplet number is depicted by the stacked circles next to the image of the lean/obese individuals. Lipid droplet–mitochondria interaction is higher in athletes vs metabolically compromised individuals. Upon endurance-exercise intervention (training depicted by the calendar), lipid droplet morphology and lipid droplet–mitochondria interactions changes towards the athlete-like phenotype in individuals who are metabolically compromised (**c**). (**d**, **e**) During an acute endurance exercise bout, fatty acids originating from lipid droplets, as well as from the circulation are used as an energy source. Endurance-trained athletes rely more heavily on IMCL to fuel exercise and have a higher lipid-droplet turnover (i.e. storage of circulation-derived fatty acids in lipid droplets and release of fatty acids originating from lipid droplets for fatty acid oxidation) than those who are metabolically compromised. This reduces the number of lipid droplets, as depicted by a smaller stack of lipid droplets in (**d**) vs (**e**). The interaction between lipid droplets and mitochondria is higher in endurance-trained athletes. This may facilitate fatty acid oxidation during exercise. Changes that occur upon exercise training in metabolically compromised individuals are shown in (**b**) and (**c**), i.e. an increased lipid droplet–mitochondrial interaction, and smaller and more lipid droplets. The hypothesised changes upon an acute exercise bout after metabolically compromised individuals have followed an endurance training intervention are represented in (**e**) and (**f**): lipid droplet–mitochondrial interaction is anticipated to increase during exercise, and lipid turnover and IMCL utilisation starts to mimic the events in athletes. Hypothetical changes are depicted using transparent illustrations. This figure is available as part of a downloadable slideset

Stable isotope measurements in combination with muscle biopsies taken before and after exercise give insights in substrate use during exercise. Both, individuals with type 2 diabetes and obese control participants mainly rely on fatty acids originating from the circulation [1, 7]. Additionally, compared with endurance-trained athletes, individuals with type 2 diabetes and obese individuals use very little IMCL as an energy source [1, 8] (Fig. 1). The lower contribution of IMCL to total fat oxidation in individuals with type 2 diabetes patients, as compared with trained individuals, may originate from dysfunctional adipose tissue and concomitant elevated plasma NEFA levels [7]. This notion is substantiated by the observation that upon acute administration of acipimox, a plasma lipid-lowering agent, the contribution of IMCL to total fat oxidation increases in type 2 diabetes patients [9]. On the other hand, it has also been observed that the contribution of IMCL to total fat oxidation was higher in trained athletes vs individuals with type 2 diabetes when matched for plasma NEFA levels [1]. This suggests that liberation of fatty acid from myocellular lipid droplets in individuals with type 2 diabetes is compromised relative to trained athletes (Fig. 1).

Myocellular lipid droplets are viewed as dynamic organelles that store and release fatty acids upon changes in energy demand and supply [10]. Lipid droplet characteristics, such as number, size, location and protein decoration, are determinants of insulin resistance [5, 11] and are remarkably different between athletes and individuals with type 2 diabetes. Unlike athletes, those with type 2 diabetes store more lipid droplets in the subsarcolemmal region [5, 8, 11] in glycolytic type II muscle fibres [5]. Lipid droplet coating proteins of the perilipin (PLIN) family play a role in lipid-droplet turnover by interacting with lipases, such as adipose triglyceride lipase (ATGL) and hormone sensitive lipase (HSL), and their co-activators. PLIN2, PLIN3 and PLIN5 are the main PLINs present in human skeletal muscle [10]. PLIN2 negatively regulates ATGL-mediated lipid droplet lipolysis by hindering access of ATGL to the lipid droplet surface [12]. PLIN3 coats nascent lipid droplets and associates with fat oxidation rates [13]. PLIN5 regulates lipolytic rate in an energy demand-dependent fashion to match fatty acid release from lipid droplets with mitochondrial fatty acid oxidation [10]. While acute exercise does not affect total PLIN5 or ATGL content [1], redistribution of PLIN5 and ATGL upon exercise to match the acute changes in energy demand may occur. Examination of the subcellular redistribution of proteins involved in myocellular lipid droplet lipolysis upon exercise has recently become possible at the level of individual lipid droplets via advanced imaging [10]. Thus, it has been shown that healthy lean participants preferentially use lipid droplets coated with PLIN2 [14, 15] and PLIN5 [14] during endurance exercise. Interestingly, the number of PLIN5-coated lipid droplets in endurance-trained athletes is higher than in individuals type 2 diabetes [6]. In addition, we observed that people with type 2 diabetes have a higher myocellular PLIN2 protein content than endurance-trained athletes [5]. Although it is commonly accepted that PLIN2 that is not bound to the lipid droplet surface is ubiquitinated and targeted for degradation, it has not yet been proven that the higher PLIN2 content in the muscle of type 2 diabetic individuals indeed implies increased decoration of the lipid droplet surface with PLIN2. Taken together, this indicates that the muscle of endurance-trained athletes is equipped for a higher exercise-mediated lipid-droplet turnover than that of individuals with type 2 diabetes. In addition, the site of lipid storage, with athletes having more lipid droplets in the intramyofibrillar area than individuals with type 2 diabetes, spatially and functionally matches a high lipid droplet-derived fat oxidative capacity. Indeed, reduction in lipid droplet number and content in the intramyofibrillar area upon acute exercise is observed [8, 16], suggesting a preferential utilisation of intramyofibrillar lipid droplets during exercise.

These studies provide novel and important insights on lipid droplet utilisation in relation to their location and protein decoration and give a better understanding of how lipid-droplet turnover is regulated during exercise in healthy individuals. This type of data, however, is lacking in individuals with type 2 diabetes. For full comprehension of why lipid droplet utilisation is compromised during endurance exercise in individuals with type 2 diabetes, a tracer study to make the distinction between whether plasma or lipid droplet-derived fatty acids are used for oxidation, along with lipid droplet-specific analysis of lipid droplet coat proteins and analysis of lipid droplet location, should be performed pre- and post-endurance exercise in individuals with type 2 diabetes.

### Lipid droplet–mitochondria interaction during endurance exercise

The more pronounced utilisation of intramyofibrillar lipid droplets during exercise may well be related to the observation that, in skeletal muscle, most lipid droplets (predominantly in the trained state, in the intramyofibrillar area) are in close proximity to mitochondria [17–19]. At the interaction sites of mitochondria and lipid droplets, there is an abundance of PLIN5 [18]. In line with the role of PLIN5 in matching lipolytic rate to fatty acid oxidation rate, PLIN5 may play a role in shuttling or chaperoning lipid droplet-released fatty acids to mitochondria for oxidation [18]. Recent studies have suggested that, when interacting with lipid droplets, mitochondria have different cellular functions than non-lipid-droplet-interacting mitochondria [19, 20]. For skeletal muscle, it has been suggested that mitochondria that are in contact with lipid droplets have a greater capacity for ATP production than non-lipid-droplet-interacting mitochondria [19]. Thus, lipid droplet–mitochondrial tethering may facilitate high fat oxidation by liberating fatty acids in the direct vicinity of mitochondria with a high capacity to oxidise fatty acids, thereby contributing to ATP maintenance during exercise. At present, experimental proof in humans for these functional processes is lacking. It should be noted, though, that trained individuals possess higher PLIN5 levels, have more PLIN5-coated lipid droplets [6] and may, thus, have more lipid droplet–mitochondrial interaction sites than individuals with type 2 diabetes. Lipid droplet–mitochondria interactions are not different between healthy lean and healthy obese participants [21, 22], but these data are lacking for individuals with type 2 diabetes in comparison with endurance-trained athletes.

Data on changes in lipid droplet–mitochondria tethering during exercise are only available for endurance-trained athletes. In male elite cross-country skiers, lipid droplet–mitochondria interactions increase upon an acute exercise bout despite unaltered IMCL content [16]. In endurance-trained women, lipid droplet–mitochondria tethering increases during exercise, with a concomitant reduction in IMCL content [23]. The latter study suggests that lipid droplet–mitochondrial interaction upon exercise promotes fatty acid oxidation. The seemingly contradictory finding that an exercise-mediated increase in lipid droplet–mitochondria interaction is paralleled by reduced IMCL content in women [23] but not in men [16] might originate from sex differences, as reviewed recently [24]. A lack of a reduction in IMCL upon exercise (as observed in the male elite cross-country skiers) may also be reflective of a high IMCL turnover (IMCL utilisation during exercise matches fatty acid incorporation into lipid droplets). The underlying mechanism for increased mitochondria–lipid droplet tethering during exercise and whether PLIN5 is important for the capacity to increase lipid droplet–mitochondrial tethering are so far unknown. Furthermore, it is not clear whether lipid droplet–mitochondrial tethering is disturbed in individuals with type 2 diabetes. The literature indicates that PLIN5 is important for lipid droplet–mitochondrial tethering [18, 20] in oxidative tissues. PLIN5 protein quantification in individual lipid droplets should be performed concomitantly with lipid droplet–mitochondrial interaction analyses in athletes and in those with type 2 diabetes upon an acute exercise bout to gain a better understanding of how lipid droplet–mitochondrial tethering works and if the capacity to tether additional mitochondria to lipid droplets upon exercise is compromised in individuals with type 2 diabetes (Fig. 1).

## Effects of exercise training on lipid metabolism in human skeletal muscle

### Mitochondrial respiratory capacity

Compromised mitochondrial respiratory capacity is frequently reported in type 2 diabetes [25–27] and obesity [26], albeit not always confirmed [28]. A potent way to increase mitochondrial respiratory capacity and a concomitant increase in fat oxidation is endurance training. Several studies have shown that mitochondrial respiratory capacity and fat oxidation increases upon endurance exercise training, even in type 2 diabetic [25, 29] and obese [25, 30] participants.

### IMCL storage, lipid droplet morphology and lipid droplet–mitochondria interactions

As well as increasing mitochondrial capacity, endurance training also is an effective intervention to improve fat oxidation and modulate fat storage in the skeletal muscle of lean sedentary participants [31]. Several studies have shown that endurance training (4–16 weeks) may affect lipid droplet characteristics without major changes in total IMCL content in type 2 diabetic [5, 11, 25, 29, 32], obese [21, 25, 33], and healthy lean, sedentary [21, 34, 35] participants. In most of these studies, however, insulin sensitivity improved. To understand this seemingly paradoxical observation, we need to focus on what happens at the lipid droplet level, rather than at the total IMCL content level. Upon exercise training, lipid droplet size [5, 22, 32] and subsarcolemmal lipid droplet content [11, 21, 22] reduces, while intramyofibrillar lipid droplet content increases [22]. These exercise-mediated changes, in previously untrained insulin-resistant individuals, resembles the IMCL storage pattern observed in insulin-sensitive endurance-trained athletes. This ‘athlete-like lipid droplet phenotype’ is characterised by many small lipid droplets in the intramyofibrillar region in type I muscle fibres with an abundance of PLIN5, and tethering of PLIN5-positive lipid droplets to mitochondria. In contrast, in individuals with type 2 diabetes, fewer but larger lipid droplets are observed, with a higher fraction of lipid droplets in the subsarcolemmal region of type II muscle fibres [5]. Lipid droplet–mitochondrial tethering increases upon endurance training in obese participants [21, 22], while no such effect was observed in individuals with type 2 diabetes [36]. All of these athlete-like changes were observed in training programmes that were carried out for more than 10 weeks (Fig. 1). Short-term training (4 weeks) in obese participants did not change lipid droplet size and number, but lipid droplet–mitochondrial interaction was increased [33]. This indicates that an athlete-like shift in lipid droplet phenotype permits storage of IMCL without impeding insulin sensitivity. A training-induced improvement in lipid droplet–mitochondrial tethering appears to be an early adaptation of endurance training that is crucial for remodelling of the IMCL storage pattern.

### IMCL utilisation/lipid-droplet turnover during endurance exercise

Training studies in healthy lean participants show that endurance training for 6 weeks promotes IMCL utilisation during exercise [14, 35, 37]. While in the untrained state PLIN2- and PLIN5-coated lipid droplets are preferentially used during exercise, 6 weeks of endurance training resulted in preferred utilisation of PLIN5-coated lipid droplets during exercise [14]. While the effect of exercise training on proteins involved in lipid-droplet turnover, such as PLIN2, PLIN5 and ATGL, has been measured, data on the effect of endurance training on IMCL utilisation and lipid-droplet turnover during an exercise bout in obese participants and individuals with type 2 diabetes is lacking (Fig. 1f). PLIN5 gene expression and protein content upon an endurance training intervention increases in obese participants and individuals with type 2 diabetes [5, 33, 38, 39]. For PLIN2 [5, 33, 38–40], PLIN3 [5, 33, 38] and ATGL [5, 38] the training effects are less consistent, either showing an increase or no change in the general population. Increased PLIN5 protein content upon endurance training indicates that IMCL use during exercise is facilitated and that lipolysis rates of lipid droplets are better matched to mitochondrial fatty acid oxidation rates in individuals with type 2 diabetes vs baseline. To test these mechanisms in a human setting, acute exercise studies in participants with type 2 diabetes are needed and should include fatty acid tracers and muscle biopsies to study IMCL utilisation during exercise, and changes in PLIN5 protein content at the lipid droplet surface before and after training. Additionally, in vitro studies in human primary myotubes obtained from endurance-trained athletes and individuals with type 2 diabetes, in combination with imaging of fatty acid tracers with live-cell imaging, can give important insights into turnover of individual lipid droplets upon exposure to different stimuli resembling exercise. Moreover, to study the direct role of PLIN5 in lipid-droplet turnover, these in vitro studies should be combined with overexpression of fluorescently tagged PLIN5 to test whether PLIN5-coated lipid droplets indeed have a higher lipid-droplet turnover.

## Exercise training and nutritional state: training in the fasted state

In most of the studies discussed above, the timing of meal intake relative to the training sessions was not monitored strictly or intentionally timed so that participants trained fasted. Interestingly, training in the (overnight) fasted state has gained popularity to promote fat oxidative capacity. Upon fasting, adipose tissue lipolysis and plasma NEFA levels increase. The increase in NEFA drives myocellular uptake of fatty acids and, thus, can promote IMCL storage and oxidation of fatty acids. Indeed, fat oxidation rates during acute exercise in the fasted state are higher than in the fed state [41, 42]. Also, the (sustained) increase in NEFA levels upon exercise in the fasted state can hypothetically provide ligands for peroxisome proliferator-activated receptor (PPAR)-mediated gene expression and, thereby, promote an adaptive response in regard to fat metabolism. Interestingly, endurance training in the fasted state improves glucose tolerance to a greater extent than training in the fed state [43]. Data on functional adaptations (like increased fat oxidative capacity) following training in the fasted state are inconsistent [35, 37, 44, 45]. Acute exercise studies measuring IMCL utilisation (with fatty acid tracers and in muscle biopsies) have been performed in the fasted state and show IMCL utilisation during exercise [1, 14, 15]. Compared with exercise in the fed state, exercising in the fasted state results in higher NEFA levels, higher fat oxidation rates and a drop in IMCL content [42]. We previously observed that, over a wide range of interventions, elevated plasma fatty acids promote IMCL storage. Whether this also occurs during exercise in the fasted state and translates into a higher flux of fatty acids in lipid droplets during exercise remains to be studied.

Upon 6 weeks of endurance training, IMCL content drops during a single exercise bout in the fasted state. This drop in IMCL content upon acute exercise was similar if the training was performed in the carbohydrate-fed state vs that fasted state [35, 37]. Currently, most training interventions under fasted conditions have only been performed in healthy lean participants and translation towards the type 2 diabetes population should be done carefully. Based on the results in healthy lean individuals, training while fasted may induce more IMCL remodelling due to a higher stimulus for lipid-droplet turnover in individuals with type 2 diabetes. Before drawing these conclusions, training interventions in the fasted vs fed state should be performed in individuals with type 2 diabetes.

## Exercise and liver lipid metabolism

**Fig. 2:**
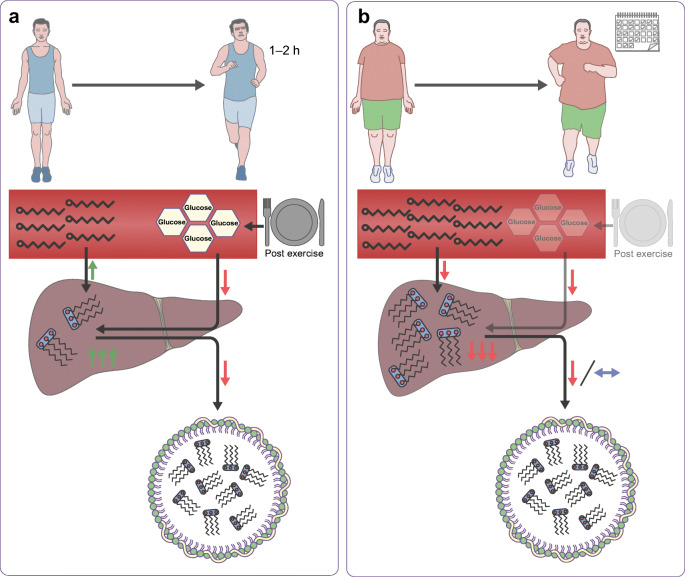
Liver lipid metabolism: acute exercise and endurance training effects. IHL content is lower in healthy lean individuals than in those who are metabolically compromised. This may be a consequence of lower plasma NEFA levels and lower rates of de novo lipogenesis in lean vs metabolically compromised individuals. (**a**) Upon acute endurance exercise, especially in the fasted state, IHL content rises, most likely due to increased plasma NEFA levels. Furthermore, VLDL-triacylglycerol secretion rates drop during acute exercise, and de novo lipogenesis is blunted due to higher postprandial glycogen synthesis by the muscle, thereby reducing glucose availability for lipid synthesis by the liver. (**b**) The underlying mechanisms that are (hypothetically) involved during endurance training in metabolically compromised individuals are shown (exercise training depicted by the calendar); these include reduced de novo lipogenesis, and improved postprandial glucose and NEFA uptake by the muscle and, thus, lower availability of glucose and NEFA for the liver to synthesise lipids. In addition, VLDL-triacylglycerol secretion rate upon endurance training in metabolically compromised individuals drops or is unchanged. Hypothetical changes are depicted using transparent illustrations. This figure is available as part of a downloadable slideset

Intrahepatic lipid (IHL) storage is associated with type 2 diabetes and cardiovascular diseases. The poor accessibility of the liver in healthy individuals means that most studies towards the effect of acute exercise and exercise training on IHLs and lipid metabolism in humans are based upon non-invasive techniques, such as MRI and tracer studies. These studies have revealed that diet and exercise both affect IHL content in obese patients with a non-alcoholic fatty liver and/or type 2 diabetes [46]. Upon endurance training for 12 weeks to 4 months, IHL content is reduced [47–49]; this has recently been extensively reviewed in *Diabetologia* [46]. Reductions in IHL content has been shown to occur in parallel with improvements in whole-body/muscle insulin sensitivity [47, 48], however, hepatic insulin sensitivity was unaffected [47] (Table 1). While a drop in IHL levels after endurance training generally occurs in the absence of changes in body weight, we observed that the training-mediated drop in IHL correlated with a drop in body fat mass [46, 47]. Increased IHL storage is, in general, not associated with disturbed VLDL-triacylglycerol secretion rates [46], and data on VLDL(-triacylglycerol) secretion rates upon endurance training is contradictory, either showing no change [49] or a decrease [50] (Table 1). It is tempting to speculate that exercise-mediated improvements in whole-body insulin sensitivity include reduced de novo lipogenesis in the liver, thereby contributing to a lower IHL content. While we are not aware of any studies underpinning this notion, it is interesting to note that a short-term (7 day) training programme resulted in altered composition (but not content) of IHL. After training, IHL contained more polyunsaturated fatty acids [51]; this is in line with lower de novo lipogenesis, which gives rise to saturated fat (Fig. 2, Table 1).

**Table 1:** Overview of results from aerobic and acute exercise training studies on parameters related to hepatic lipid metabolism and whole-body glucose homeostasis

Study	Group	Acute exercise or training	Protocol	Results relating to hepatic parameters	Results relating to glucose homeostasis
Alam et al, 2004 [50]	T2D	Supervised aerobic exercise	6 months; 4 × per week; 20–40 min at 60–85% $\dot{V}{\mathrm{O}}_{2\max }$	VLDL-ApoB-100 pool size ↓ VLDL-ApoB-100 secretion rate ↓	HbA_1c_ ↓ IS ↑
Bacchi et al, 2013 [48]	T2D with NAFL	Aerobic or resistance training	4 months; 3 × per week Aerobic: 60 min/session at 60–65% HRR on treadmill, cycle or elliptical machine Resistance: 9 different exercises involving major muscle groups on weight machines	IHL ↓	HbA_1c_ ↓ IS ↑
Bilet et al, 2015 [41]	Obese	Acute aerobic exercise	2 h cycling in fed and fasted state at 50% *W*_max_	IHL 30 min post-exercise ↔ IHL 4 h post-exercise, fed state ↔ IHL 4 h post-exercise, fasted state ↓	ND
Brouwers et al, 2018 [47]	Obese control and obese NAFL	Supervised combined endurance and resistance training	12 weeks Endurance: 2 × per week; cycling for 30 min at 70% $\dot{V}{\mathrm{O}}_{2\max }$ Resistance: 1 × per week; 3 sets of 10 repetitions at 60% MVC of large muscle groups	IHL ↓	EGP ↔ Skeletal muscle IS ↑ NOGD ↑ Glucose oxidation ↔
Egger et al, 2013 [52]	Healthy lean	Acute aerobic exercise	2 h treadmill walking at 50% $\dot{V}{\mathrm{O}}_{2\max }$	IHL ↑	ND
Haus et al, 2013 [51]	Obese NAFL	Short-term aerobic training	7 consecutive days; 60 min treadmill walking at ~85% HR_max_	Hepatic PUI ↑	IS ↑
Rabol et al, 2011 [54]	Young, insulin resistant, lean	Acute aerobic exercise	45 min on elliptical trainer: 3 sets of 15 min at 70–85% HR_max_	Postprandial de novo lipogenesis ↓ Postprandial hepatic TG synthesis ↓	ND
Sondergaard et al, 2011 [53]	Healthy lean	Acute aerobic exercise	1.5 h moderate-intensity exercise	VLDL-TG concentration ↔ Non-VLDL-TG concentration ↓ VLDL-TG secretion rates ↓ Contribution of VLDL-TG oxidation to total energy expenditure ↓	ND
Sullivan et al, 2012 [49]	Obese NAFL	Aerobic training	16 weeks; 5 × per week (1 × supervised, 4 × at home); 30–60 min brisk walking (45–55% $\dot{V}{\mathrm{O}}_{2\mathrm{peak}}$)	IHL ↓ VLDL-TG secretion rates ↔ VLDL-ApoB-100 secretion rate ↔	ND

Apo, apolipoprotein; EGP, endogenous glucose production; HR_max_, maximal heart rate; HRR, heart rate reserve; IS, insulin sensitivity; MVC, maximal voluntary contraction; NAFL, non-alcoholic fatty liver; ND, not determined; NOGD, non-oxidative glucose disposal; PUI, polyunsaturated lipid index; T2D, type 2 diabetes; TG, triacylglycerol

As exercise training reduces IHL content [47, 48], one could suggest that IHL also drops upon acute exercise. We observed that, upon 2 h of endurance exercise, IHL content was unaffected, irrespective of participants being in the fed or fasted stated. After exercise and upon recovery in the fasted state, however, we observed an increase in IHL [41]. Additionally, IHL increases upon an exercise bout in active lean participants who consumed a light meal before the start of the exercise [52]. Interestingly, in both studies [41, 52], increased IHL content after exercise occurred in the presence of elevated plasma NEFA levels. If this rise in plasma NEFAs is prevented by providing a glucose drink every half hour during and after exercise, IHL does not increase. This indicates that the rise in plasma NEFA levels upon exercise drives the increased IHL content after an exercise bout.

IHL can be used during exercise, upon secretion of VLDL-triacylglycerols into the bloodstream. VLDL-triacylglycerol kinetic analyses during an acute exercise bout in the fasted state show that VLDL-triacylglycerol secretion rates drop during exercise and that the contribution of these particles to total energy expenditure is decreased [53]. Thus, besides the increase in NEFA influx, the lower VLDL-triacylglycerol secretion rates during exercise may also contribute to the increase in IHL content after acute exercise in the fasted state (Fig. 2). In lean, normoglycaemic but insulin-resistant individuals, postprandial IHL synthesis and de novo lipogenesis is lower after a single bout of exercise compared with rest [54].

Overall, IHL may increase upon acute exercise, but is lower after training, possibly due to lower postprandial de novo lipogenesis during recovery. It is also lower in endurance-trained individuals. It is currently unknown how the apparent increase in IHL after acute exercise turns into reduced IHL content after endurance training. We cannot exclude that training, per se, is not the major determinant of IHL but that the dietary habits of trained individuals may also make an important contribution.

## Concluding remarks

IMCL and IHL content are increased, and fat oxidative capacity decreased in metabolically compromised individuals, such as obese individuals and those with type 2 diabetes. While endurance exercise training reduces total intracellular fat content in the liver, the effects in muscle indicate remodelling rather than lowering of the myocellular lipid droplet pool. In fact, in most populations and under most conditions, endurance exercise training augments IMCL content. Thus, the ability of exercise to modulate lipid droplet dynamics in the liver and muscle contributes to differences in fat oxidative metabolism. Endurance training in individuals with type 2 diabetes remodels IMCL content towards an athlete-like phenotype, while IHL content is reduced. While many training intervention studies have been performed in metabolically compromised individuals, the effects of acute exercise have not been extensively studied, particularly not in participants with type 2 diabetes. Thus, it is unclear why IMCL utilisation during exercise is lower in individuals with type 2 diabetes and whether the observed IMCL remodelling towards the athlete-like phenotype in these individuals also translates into the anticipated increase in IMCL utilisation during exercise. Study findings on the effects of sex differences and exercise intensity on IMCL use during exercise or lipid droplet remodelling upon training are either contradictory or lacking. Compared with skeletal muscle, the underlying mechanisms of the effects of exercise and training on IHL are even more poorly understood. The reduction in IHL content upon training that is observed in metabolically compromised individuals may partly originate from reduced postprandial de novo lipogenesis. Since diurnal rhythms are present in lipid metabolism, future studies should also focus on the effect of timing of exercise on the parameters discussed in this review in order to elucidate the optimal conditions for exercise-induced improvements in insulin sensitivity in individuals with type 2 diabetes.

## Electronic supplementary material

Slideset of figures (PPTX 842 kb)

## Abbreviations

ATGL: Adipose triglyceride lipase
IHL: Intrahepatic lipid
IMCL: Intramyocellular lipid
PLIN: Perilipin
*W*_max_: Maximal power output
