# Supplementary material for: Exercising your fat (metabolism) into shape: a muscle-centred view
Source: Diabetologia. 2020 Jun 12;63(8):1453–63. doi: 10.1007/s00125-020-05170-z (PMC7351830; doi:10.1007/s00125-020-05170-z)
Supplement: Supplementary file 1 — (PPTX 842 kb) [file 125_2020_5170_MOESM1_ESM.pptx]

## Slide 1
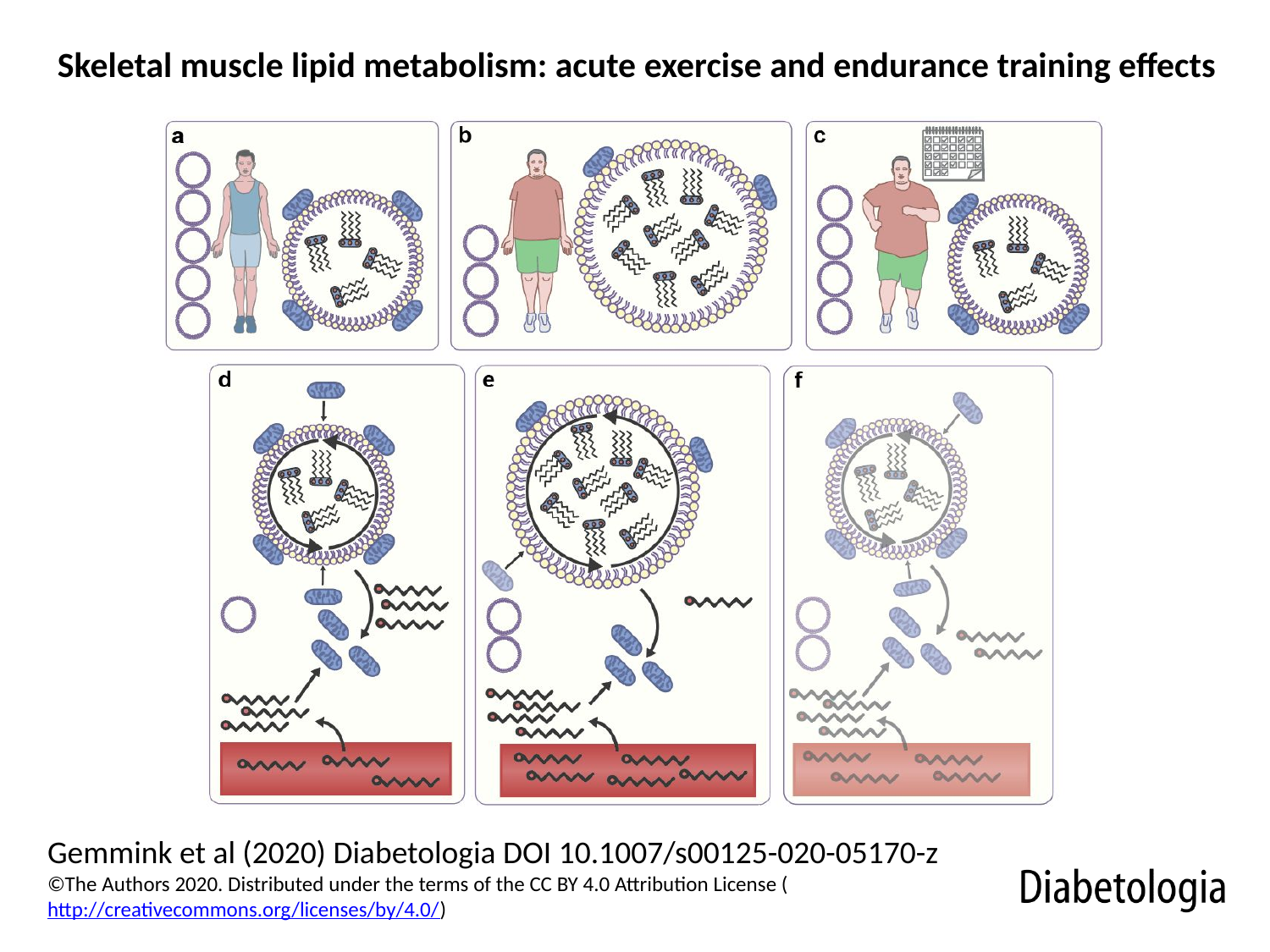

Skeletal muscle lipid metabolism: acute exercise and endurance training effects
Gemmink et al (2020) Diabetologia DOI 10.1007/s00125-020-05170-z
©The Authors 2020. Distributed under the terms of the CC BY 4.0 Attribution License (http://creativecommons.org/licenses/by/4.0/)

## Slide 2
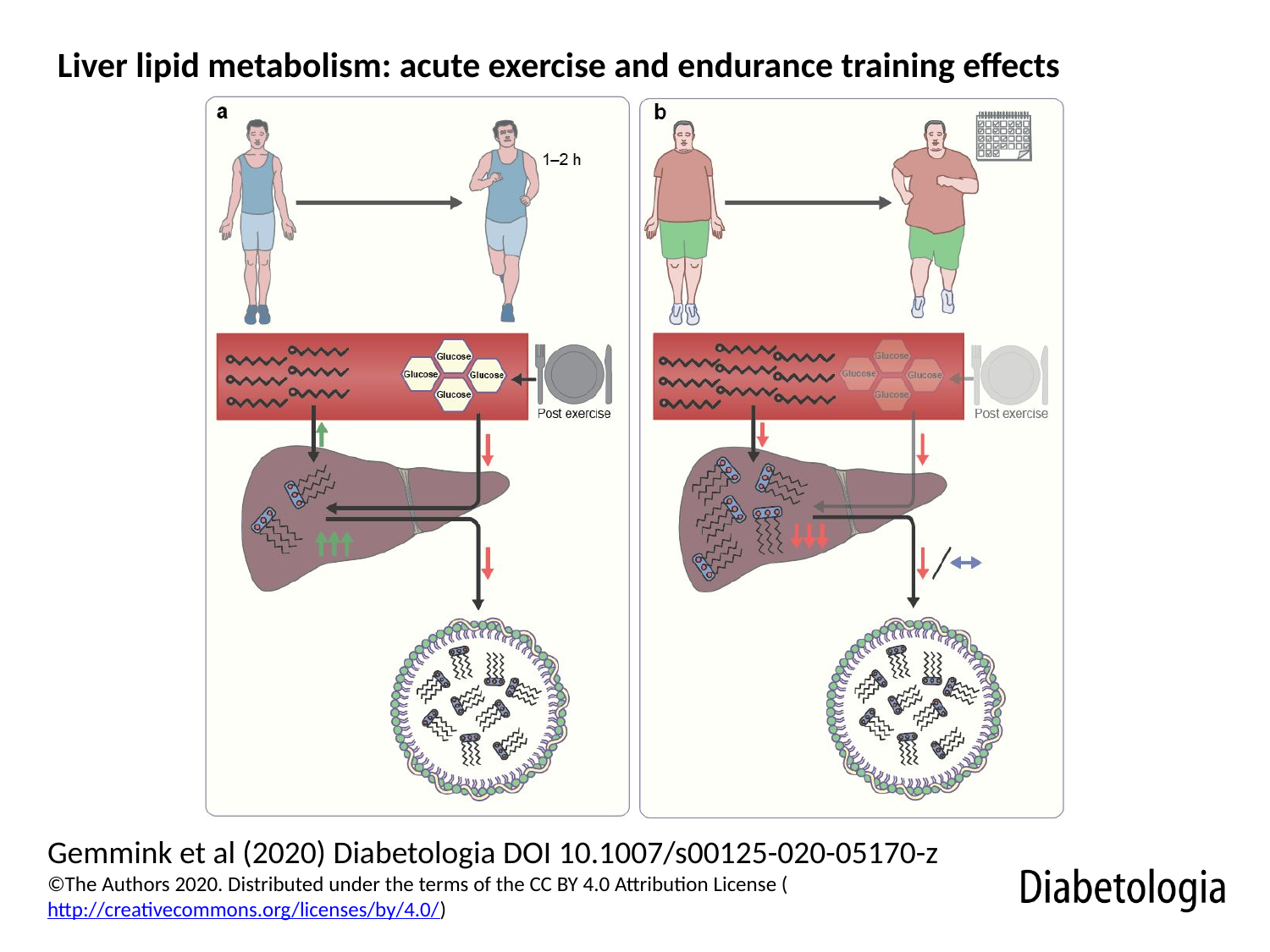

Liver lipid metabolism: acute exercise and endurance training effects
Gemmink et al (2020) Diabetologia DOI 10.1007/s00125-020-05170-z
©The Authors 2020. Distributed under the terms of the CC BY 4.0 Attribution License (http://creativecommons.org/licenses/by/4.0/)
